# The retinoid signalling molecule, TRIM16, is repressed during squamous cell carcinoma skin carcinogenesis *in vivo* and reduces skin cancer cell migration *in vitro*

**DOI:** 10.1002/path.2986

**Published:** 2011-10-18

**Authors:** Belamy B Cheung, Jessica Koach, Owen Tan, Patrick Kim, Jessica L Bell, Carla D'andreti, Selina Sutton, Alena Malyukova, Eric Sekyere, Murray Norris, Michelle Haber, Maria Kavallaris, Anne M Cunningham, Charlotte Proby, Irene Leigh, James S Wilmott, Caroline L Cooper, Gary M Halliday, Richard A Scolyer, Glenn M Marshall

**Affiliations:** 1 Children's Cancer Institute Australia for Medical Research, Lowy Cancer Research Centre, University of NSWRandwick, NSW, Australia; 2 School of Women's and Children's Health, Faculty of MedicineUNSW, Australia; 3 University of DundeeDundee, UK; 4 Tissue Pathology and Diagnostic Oncology, Royal Prince Alfred Hospital, Sydney and Discipline of Pathology, Central Clinical School, The University of SydneyAustralia; 5 Melanoma Institute AustraliaSydney, Australia; 6 Dermatology, Bosch Institute, University of SydneyAustralia; 7 Centre for Children's Cancer and Blood Disorders, Sydney Children's HospitalRandwick, New South Wales, Australia

**Keywords:** TRIM16, tripartite motif protein, squamous cell carcinoma, vimentin, E2F1

## Abstract

Retinoid therapy is used for chemo-prevention in immuno-suppressed patients at high risk of developing skin cancer. The retinoid signalling molecule, tripartite motif protein 16 (TRIM16), is a regulator of keratinocyte differentiation and a tumour suppressor in retinoid-sensitive neuroblastoma. We sought to determine the role of TRIM16 in skin squamous cell carcinoma (SCC) pathogenesis. We have shown that TRIM16 expression was markedly reduced during the histological progression from normal skin to actinic keratosis and SCC. SCC cell lines exhibited lower cytoplasmic and nuclear TRIM16 expression compared with primary human keratinocyte (PHK) cells due to reduced TRIM16 protein stability. Overexpressed TRIM16 translocated to the nucleus, inducing growth arrest and cell differentiation. In SCC cells, TRIM16 bound to and down regulated nuclear E2F1, this is required for cell replication. Retinoid treatment increased nuclear TRIM16 expression in retinoid-sensitive PHK cells, but not in retinoid-resistant SCC cells. Overexpression of TRIM16 reduced SCC cell migration, which required the C-terminal RET finger protein (RFP)-like domain of TRIM16. The mesenchymal intermediate filament protein, vimentin, was directly bound and down-regulated by TRIM16 and was required for TRIM16-reduced cell migration. Taken together, our data suggest that loss of TRIM16 expression plays an important role in the development of cutaneous SCC and is a determinant of retinoid sensitivity.

Copyright © 2011 Pathological Society of Great Britain and Ireland. Published by John Wiley & Sons, Ltd.

## Introduction

Squamous cell carcinomas (SCCs) can occur in any anatomic site where squamous cells exist, including the skin and squamous-lined mucosal membranes. Actinic keratosis (AK) and Bowen's disease (also termed SCC *in situ*) are the two primary precursors of cutaneous SCC 1. In addition to producing destructive local growth, SCC may invade nerves, lymphatics, and blood vessels, leading to locoregional or distant metastasis. Whilst precursor lesions (AK and Bowen's disease) may be treated by topical therapies, invasive SCC usually requires surgical excision. Surgery is usually well tolerated, but occasionally may result in significant morbidity, wound infection, and excessive scarring. Chronic solar ultraviolet radiation exposure is the most important risk factor for the development of SCC, inducing DNA damage which can lead to keratinocyte transformation and modification of the cutaneous immune response 2, 3. Understanding the molecular alterations that underlie SCC may allow the identification of novel therapeutic targets and facilitate the development of rational therapies.

Retinoids are vitamin A analogues which induce growth arrest, cell death, and differentiation in cancer cells *in vitro* when used in pharmacological doses 4, 5. In sensitive cancer cell types, retinoids entrain terminal tissue differentiation programmes, and this feature has been used therapeutically in patients. In cancer patients, retinoids are most effective in states of minimal residual disease and have low side-effect profiles when compared with conventional chemotherapeutic agents 6, 7. Retinoids have been used for the chemoprophylaxis of skin cancers in susceptible individuals 8. Retinoids are effective for the treatment of actinic keratoses and delay the development of SCCs in patients with xeroderma pigmentosum, an inherited predisposition to ultraviolet-induced cancer 8, 9. However, the factors which determine the selective action and sensitivity of retinoids in cancer cells compared with normal cells have not yet been fully elucidated.

The TRIM16 (or oestrogen-responsive B box protein, EBBP) gene is located on chromosome 17p11.2. It is associated with many different kinds of cancers 10–12. Analysis of the TRIM16 gene product has found that it is a member of the tripartite motif (TRIM) protein family (also known as the RBCC family). This protein family is characterized by three zinc-binding domains, a RING, a B-box type 1, and a B-box type 2, followed by a coiled-coil region 13, 14. Some TRIM proteins homo-multimerize through their coiled-coil region, and the integrity of this TRIM motif is required for proper subcellular localization of TRIM proteins. In searching for factors that mediate the retinoid anti-cancer signal, we have identified TRIM16 as a DNA binding protein with histone acetyltransferase activity, which is necessary for the retinoic acid receptor β_2_ transcriptional response in retinoid-treated cancer cells 15, 16. We demonstrated that overexpressed TRIM16 reduced neuroblastoma cell growth, enhanced retinoid-induced differentiation, and decreased tumourigenicity *in vivo*. Our studies revealed that TRIM16 acts as a tumour suppressor, promoting neuritic differentiation, cell migration, and replication through interactions with cytoplasmic vimentin and nuclear E2F1 in neuroblastoma cells 17.

In this study, we sought to elucidate the molecular function of TRIM16 in skin carcinogenesis. We found decreased expression of TRIM16 in human skin SCC in comparison to normal skin. Retinoid treatment increased the nuclear level of TRIM16 in human primary keratinocytes, but not in SCC cells. We also discovered that overexpression of TRIM16 inhibited cell growth and cell migration by down-regulating E2F1, pRb phosphorylation, and vimentin in SCC cells. Moreover, TRIM16 suppressed cell migration through its RFP domain. Our results thus provide novel evidence which supports the role of TRIM16 in cell proliferation, migration, and differentiation in cutaneous SCC.

## Materials and methods

### Immunohistochemistry of patient tissue samples

Following Institutional Review Board Human Research Ethics Committee approval (Protocol No X06-0306), archival formalin-fixed, paraffin-embedded tissue blocks of excised human skin specimens were retrieved from the Department of Tissue Pathology and Diagnostic Oncology at the Royal Prince Alfred Hospital, Sydney, Australia. Five-micrometre-thick tissue sections were cut from the paraffin blocks and placed on saline-coated slides. The sections were incubated with a custom-made rabbit anti-TRIM16 (sequence of the peptides: Ac-CTNTTPWEHPYPDLPS-amide) (Invitrogen, Victoria, Australia) or rabbit IgG (I-1000; Vector Laboratories, Burlingame, CA, USA) at 1.91 µg/µl at 4 °C overnight. Sections were then incubated with secondary goat anti-rabbit immunoglobulin/biotinylated (E0432; Dako, Glostrup, Denmark) at 1 in 500 dilution for 1 h at room temperature.

### Analysis of immunohistochemistry of skin tissue samples

Eight tissues were selected based on their TRIM16 staining intensity to construct a reference range for intensity. A semi-quantitative score was assigned to each level of staining: 0 for negative staining, 1 for weak staining, 2 for weak–moderate staining, 3 for moderate–strong staining, and 4 for strong staining. Each patient slide was then scored by a blinded researcher based on the intensity reference range. Four fields per patient sample were observed and the average score was recorded. Statistical analysis was performed using one-way analysis of variance.

### Cell culture

Primary human keratinocytes (PHKs) were purchased from Gibco (Victoria, Australia) and cultured in Defined Keratinocyte-SFM. The PHKs were obtained from a pool of four neonatal foreskins. Tumourigenic keratinocytes (SCC-15), derived from squamous cell carcinoma, and HEK001, proliferating basal-type non-malignant keratinocytes and transformed by human papillomavirus 16 (HPV-16) E6/E7, were purchased from ATCC (Manassas, VA, USA) and cultured in DMEM/F12 (1 : 1) medium containing 10% fetal calf serum (FCS) and 0.4 µg/ml hydrocortisone. A skin SCC cell line, HSC-1, was purchased from the NIHS (JCRB, Osaka, Japan) Cell Bank and cultured in DMEM with 10% FCS. The human keratinocyte cell line, HaCaT cells, was grown in DMEM with 10% FCS. Two SCC lines (MET-1 and MET-4) derived from clinical progression of a primary epidermal tumour through to distant metastasis were cultured in DMEM/F12 (3 : 1) medium containing 10% FCS and a selection of growth hormones as previously described 18.

### Immunoprecipitation assays and western blots

Lysates were immunoprecipitated with anti-Turbo GFP (Evrogen, Moscow, Russia). Anti-vimentin antibody and cytokeratin 1/10 (Santa Cruz Biotechnology, Inc, Santa Cruz, CA, USA), anti-E2F1 antibody (Cell Signaling, Danvers, MA, USA), anti-cyclin E2, anti-involucrin (Sigma, Sydney, Australia), anti-myc tag antibody, anti-Flag tag antibody (Cell Signaling), and anti-TRIM16 (Bethyl Laboratories, TX, USA) were used in the immunoblots. Rabbit polyclonal actin antibody (Sigma, St Louis, MO, USA), and histone H3 antibody and anti-GAPDH antibody (Cell Signaling) were used to normalize for differences in whole cell lysates, nuclear or cytoplasmic protein loading, respectively. Western blot band intensity was measured using a scanning densitometer (Bio-Rad, Australia) and Quantity One imaging software (Bio-Rad). Each protein band was normalized to its actin counterpart for loading control.

Detailed experimental procedures can be found in the Supporting information, Supplementary materials and methods.

## Results

### Reduced expression of TRIM16 in human skin SCC compared with normal skin

**Figure 1:**
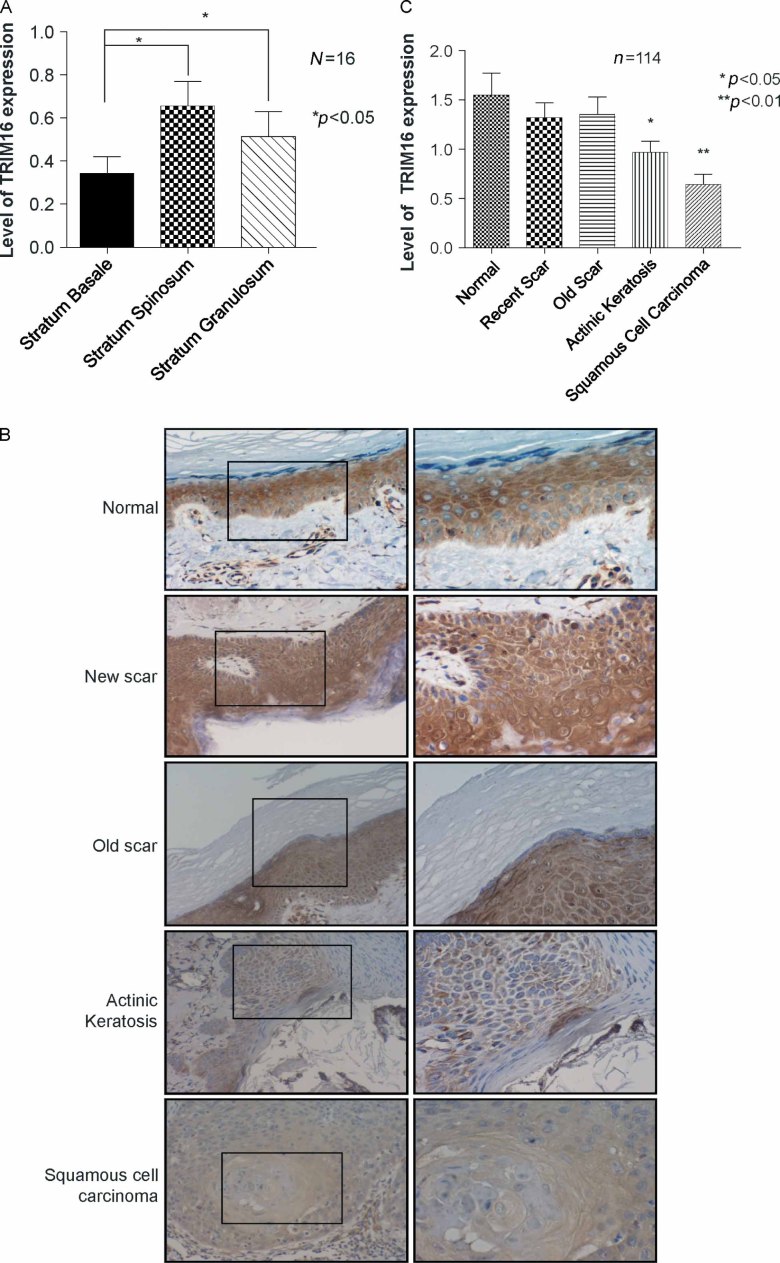
Reduced expression of TRIM16 in human skin SCC compared with normal skin. (A) The distribution of TRIM16 expression in normal epidermis: the data were collected from 16 normal patient skin samples; four fields were selected from each sample. (B) Representative sections showing immunohistochemical staining for TRIM16 expression (brown staining) in five categories of primary human skin tissues, using an anti-TRIM16 antibody (scale bar = 100 µm). Original magnification: left panels, 200×; right panels, 400×. (C) Analysis of the staining intensity of 114 patients showed that TRIM16 expression is markedly reduced during the progression from normal skin to SCC. Statistical analysis was performed using one-way analysis of variance

It has been reported that the presence of high levels of TRIM16 in keratinocytes is important for the onset of keratinocyte differentiation under permissive conditions 19. Thus, TRIM16 could play an important role in the induction of the differentiation pathway and be involved in skin cancer progression. We therefore examined the expression level of TRIM16 protein by immunohistochemical staining using a TRIM16-specific antibody in 114 surgically excised skin tissue samples. The skin specimens were classified into the following five categories on the basis of their morphological features by two pathologists (CLC and RAS)—normal skin (16 patients), recent scars (<30 days old; 28 patients), old scars (OC) (>30 days old; 27 patients), actinic keratosis (27 patients), and SCC (16 patients)—and scored according to the grading of staining for TRIM16 expression. TRIM16 was most strongly expressed in the cytoplasm and nucleus of the stratum granulosum and stratum spinosum of the epidermis, compared with the stratum basale layer in normal skin (Figure 1A), indicating that TRIM16 is expressed as keratinocytes stop dividing and commence differentiation. Conversely, TRIM16 expression was reduced in actinic keratoses and further diminished in SCC (Figure 1B), consistent with a lack of expression in dividing, poorly differentiated keratinocytes. Analysis of the staining intensity showed that TRIM16 expression was markedly reduced during the progression from normal skin to SCC (Figure 1C). There was no significant difference in TRIM16 expression in the epidermis overlying healed scar tissue compared with normal skin, showing that TRIM16 levels were normal upon completion of wound healing.

### TRIM16 protein is regulated by the proteasome-dependent pathway and its expression level is reduced in SCC cells

**Figure 2:**
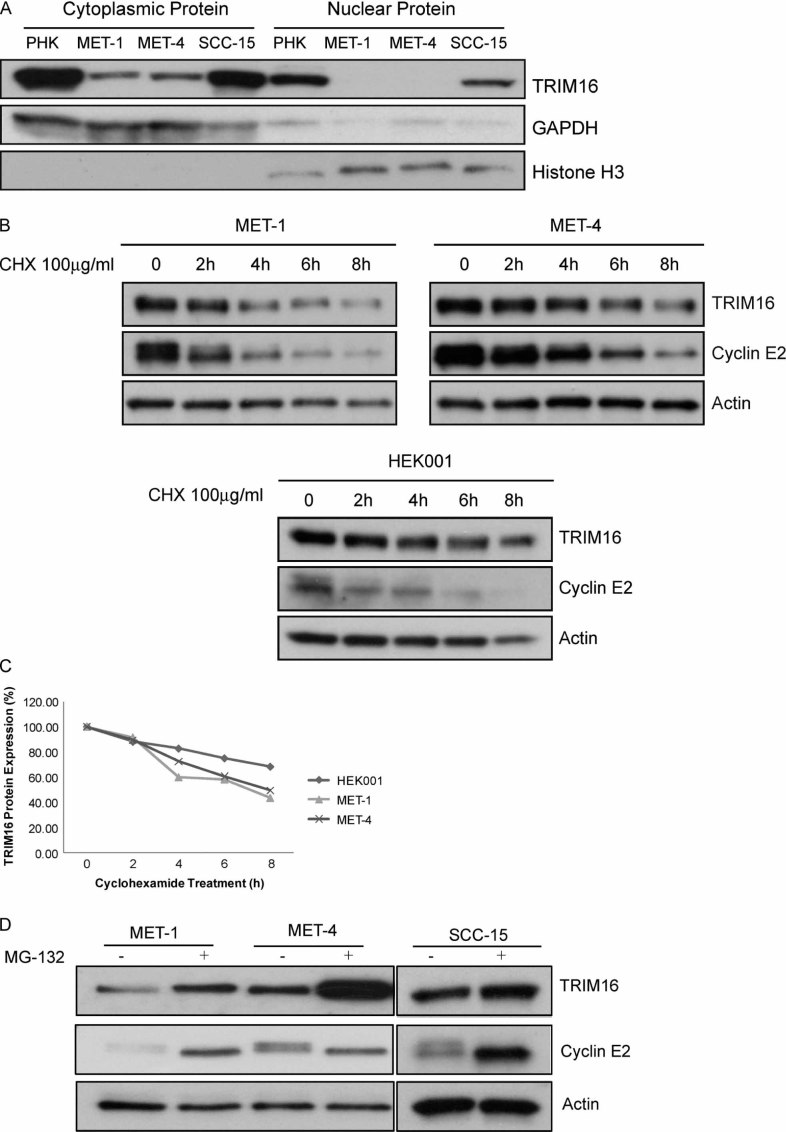
TRIM16 protein is regulated by the proteasome-dependent pathway and its expression level is reduced in SCC cells. (A) Immunoblotting analysis of the expression of cytoplasmic and nuclear proteins TRIM16 in PHKs, MET-1, MET-4, and SCC-15 cells. Anti-histone H3 was used as a control for nuclear protein expression, and the anti-GAPDH antibody as a cytoplasmic protein control. (B, C) MET-1, MET-4, and HEK001 cells were treated with cycloheximide at a final concentration of 100 µg/ml over 8 h. At the specified time points, the cells were harvested and the protein was extracted for analysis by western blots. The western blots were probed with anti-cyclin E2 and anti-actin antibodies as controls. (D) Cells were treated with 30 µm MG-132 for 5 h. Whole cell lysates were prepared and western blots were probed with anti-TRIM16, cyclin E2, and actin antibodies

Endogenous and recombinant TRIM16 are mainly localized to the cytoplasm of COS-1 cells (monkey kidney cells) and HaCaT keratinocytes 19. Here we sought to investigate TRIM16 cellular localization in both PHKs and SCC cells. First, we measured *TRIM16* mRNA by real-time PCR and found similar TRIM16 expression levels in PHKs and three SCC cell lines (data not shown). In contrast, western blot showed that nuclear protein expression of TRIM16 was markedly decreased in MET-1, MET-4, and SCC-15 cells, and the cytoplasmic expression of TRIM16 was also decreased in MET-1 and MET-4 cell lines, compared with normal PHKs (Figure 2A). Next, we used the protein synthesis inhibitor cycloheximide to determine the half-life of the TRIM16 protein in SCC cells. We observed that the TRIM16 half-life in MET-1 and MET-4 cells was 7–7.5 h, compared with a projected TRIM16 half-life in HEK001 cells of 12 h (Figures 2B and 2C). Cyclin E2 expression was used as a positive control for the cycloheximide treatment. After the cells were treated with the proteasome inhibitor MG-132 for 5 h, we found markedly increased TRIM16 in MET-1 and MET-4 cells, and slightly increased TRIM16 in SCC-15 cells (Figure 2D). These data suggest that TRIM16 protein stability is reduced in SCC cells and that degradation occurs by the proteasome-dependent pathway.

### Retinoid treatment increases nuclear TRIM16 in PHKs, but not in SCC cells

**Figure 3:**
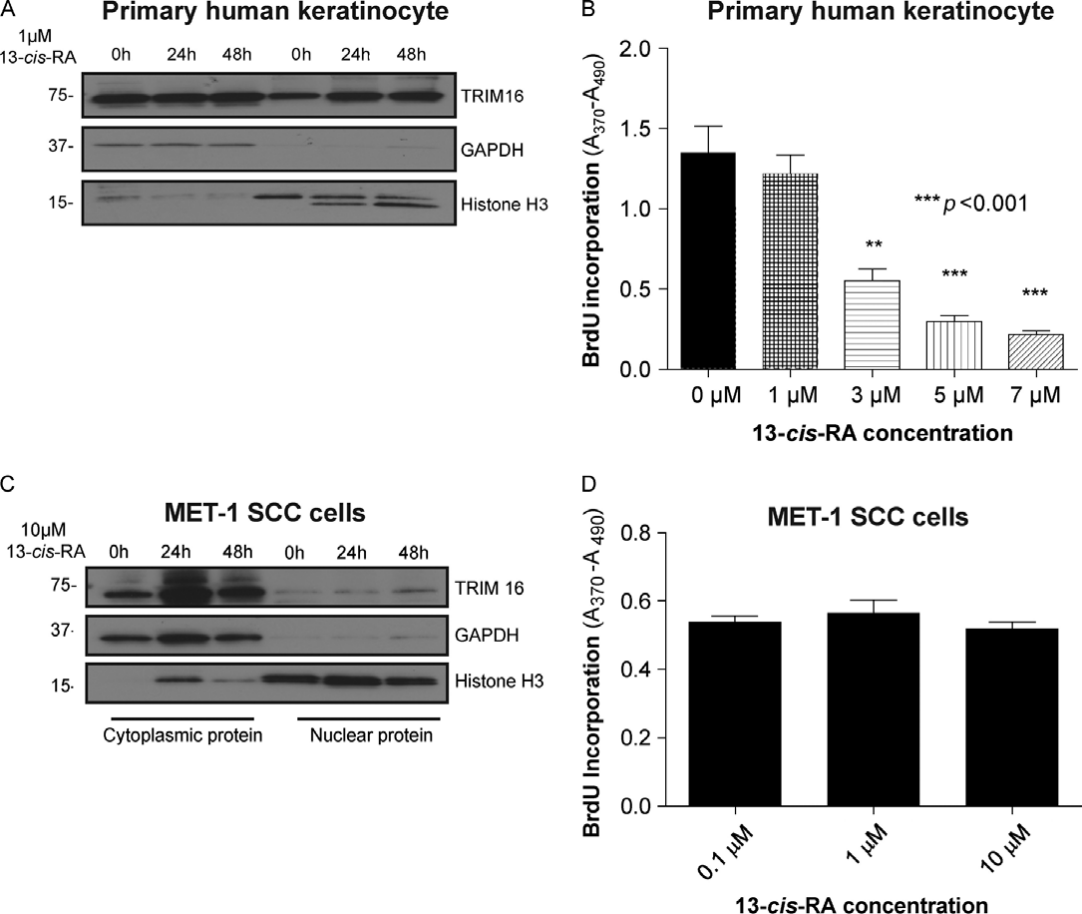
Retinoid treatment increases the nuclear level of TRIM16 in PHKs, but not in SCC cells. (A, C) Immunoblotting analysis of cytoplasmic and nuclear TRIM16 proteins in PHKs and MET-1 cells treated with 1 µm 13-*cis*-RA or 10 µm 13-*cis*-RA for 24 and 48 h, respectively. Anti-histone H3 was used as a control for nuclear protein, and anti-GAPDH antibody as a cytoplasmic protein control. (B, D) PHKs and MET-1 cells were treated with the indicated concentrations of 13-*cis*-RA for 48 h, followed by incubation with BrdU for the last 6 h. BrdU incorporation was measured as OD units of absorbance

TRIM16 expression is increased by oestrogen and keratinocyte growth factor in skin cells 19, and we have also shown that retinoid treatment causes TRIM16 to translocate to the nucleus 17. Here we sought to investigate whether retinoid has effects on TRIM16 cellular localization in skin cells. PHKs were treated with 1 µm 13-*cis*-retinoic acid (13-*cis*-RA). We found that the nuclear level of TRIM16 was increased at both 24 and 48 h compared with untreated cells (Figure 3A), and cell proliferation was significantly reduced by 3 µm 13-*cis*-RA treatment (Figure 3B). In contrast, treatment with up to 10 µm 13-*cis*-RA did not change the TRIM16 cytoplasmic or nuclear levels, or cell proliferation in MET-1 cancer cells (Figures 3C and 3D). Since TRIM16 has been demonstrated to sensitize cancer cells to retinoids in neuroblastoma and breast cancer cells 15, 16, the loss of endogenous nuclear TRIM16 may be one possible cause of retinoid resistance in skin SCC cells.

### Increased TRIM16 expression induces differentiation in SCC cells

**Figure 4:**
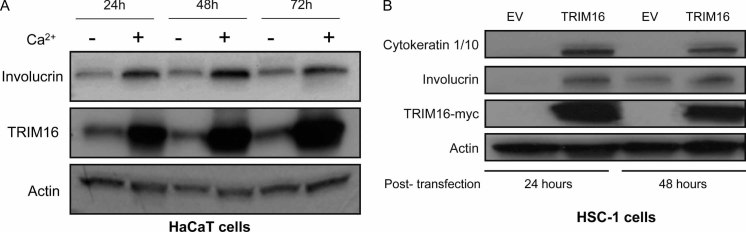
Increased TRIM16 expression induces differentiation in SCC cells. (A) Differentiated HaCaT cells were maintained in the presence of Ca^2+^ (+), and dedifferentiated cells in the absence of Ca^2+^ (−). Whole cell lysates of the HaCaT cells were subjected to the immunoblotting for involucrin, TRIM16, or actin. (B) Immunoblots of total cellular proteins from confluent HSC-1 cells transiently overexpressing TRIM16 plasmid DNA and control cells transfected with empty vector using cytokeratin 1/10, involucrin, myc-tag, and actin antibodies

To address the question of whether TRIM16 expression plays a role in keratinocyte differentiation, we used the HaCaT differentiation model 20. HaCaT cells are immortalized human normal skin keratinocytes and can recapitulate keratinocyte differentiation *in vitro*. In the presence of added calcium (Ca^2+^), HaCaT cells express involucrin 20. Untreated HaCaT cells expressed TRIM16, but expression was markedly increased following the addition of calcium to the culture medium (Figure 4A). Next, we transiently transfected TRIM16 plasmid DNA into HSC-1 SCC cells; overexpression of TRIM16 increased the expression level of cytokeratin 1/10 and involucrin, both differentiation markers, in HSC-1 cells (Figure 4B). These results suggest that TRIM16 may play a role in the cell differentiation of skin cancer cells.

### Enforced overexpression caused TRIM16 translocation to the nucleus, reduced cell growth, and decreased nuclear E2F1 and pRb phosphorylation

**Figure 5:**
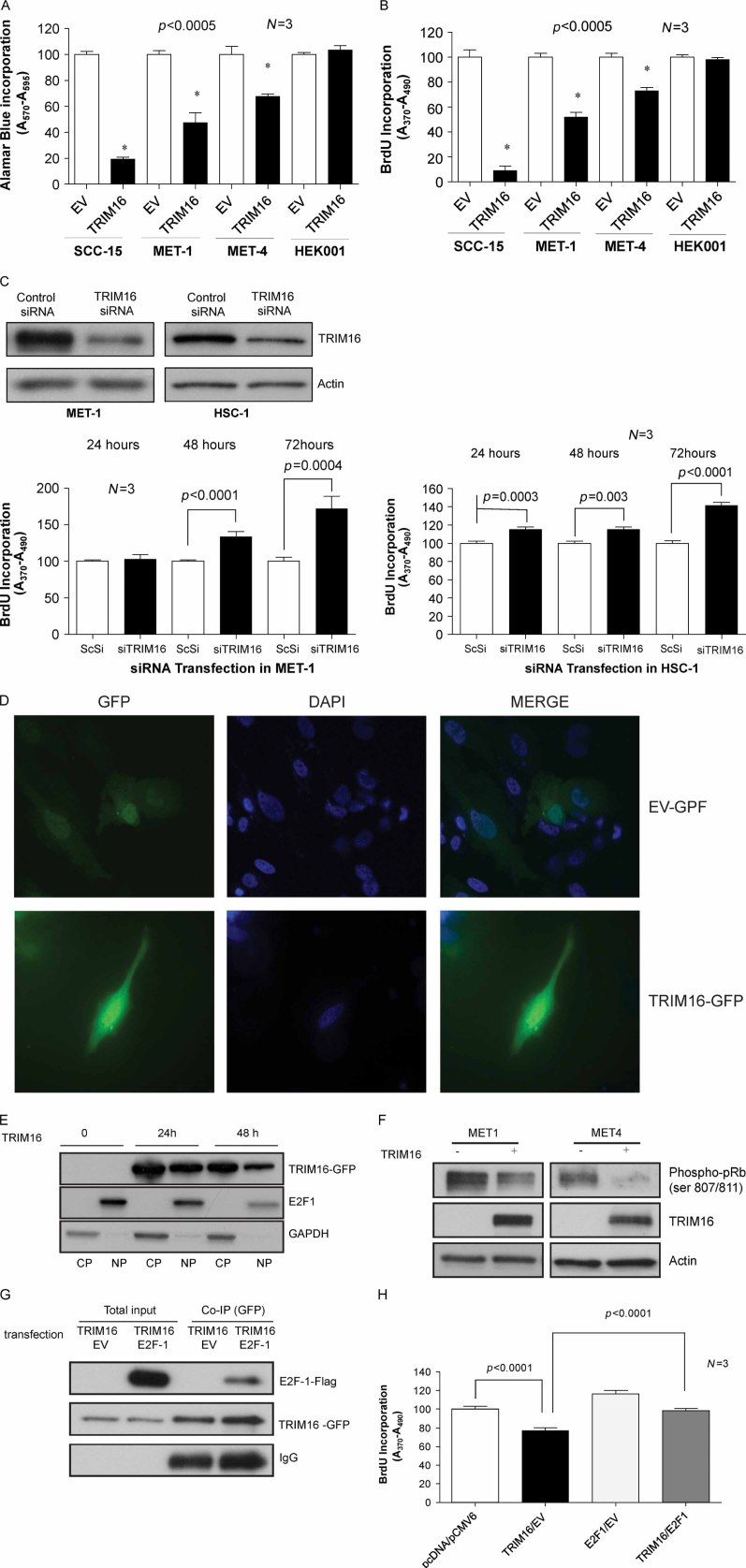
Enforced overexpression caused TRIM16 translocation to the nucleus, reduced cell growth, and decreased nuclear E2F1 and pRb phosphorylation. (A, B) Cell viability and proliferation were measured at 72 h by Alamar Blue assay or BrdU incorporation, respectively. Cells were transiently transfected with empty vector (EV) or with TRIM16 plasmid DNA. (C) Top panel: western blotting analysis by TRIM16-specific antibody at 72 h of MET-1 and HSC-1 cells with control siRNA or TRIM16 siRNA transfection. Bottom panel: cell proliferation was measured up to 72 h by BrdU incorporation assay in MET-1 and HSC-1 cells transfected with control siRNA (ScSi) or TRIM16-specific siRNA (siTRIM16). (D) Immunofluorescence microscopy of nuclear TRIM16 staining in MET-1cells after empty vector and GFP-TRIM16 transfection for 24 h. TRIM16 protein was detected with fluorescein (green), and nuclei were identified with DAPI (blue). (E) Immunoblotting analysis of the expression of TRIM16 and E2F1 in MET-1 cells. Cytoplasmic (CP) and nuclear (NP) proteins of cells transfected with TRIM16 plasmid DNA for 24 and 48 h were analysed by immunoblotting using anti-TRIM16 and anti-E2F1 antibodies. (F) Phospho-pRb (ser807/811) protein was analysed by western blot with samples from MET-1 and MET-4 cells, transiently transfected with TRIM16 cDNA plasmid or empty vector. Actin was used as a loading control. GFP-tag antibody was probed for confirmation of TRIM16 plasmid transfection. (G) Lysates of MET-1 cells transfected with the indicated plasmids (lanes 1 and 3: GFP-TRIM16 and pCMV-6 empty vectors; lanes 2 and 4: GFP-TRIM16 and pCMV-6-E2F1) were immunoprecipitated by anti-GFP antibody and analysed by immunoblotting using anti-Flag and anti-GFP antibodies. (H) MET-1 cells were transfected with different plasmid DNAs for 72 h, followed by incubation with BrdU for the last 6 h

To determine whether TRIM16 plays a role in skin cancer cell growth, we transiently transfected human TRIM16 plasmid DNA into three different SCC cell lines. Transient overexpression of TRIM16 led to a significant decrease in viable SCC-15, MET-1, and MET-4 cells when compared with empty vector control (*p* < 0.0005), as measured by the Alamar Blue assay at 48 h after transfection (Figure 5A). In cell proliferation studies, BrdU incorporation in SCC-15, MET-1, and MET-4 cells was decreased in cells transfected with TRIM16 plasmid DNA, compared with the empty vector control (*p* < 0.0005) (Figure 5B). In contrast, overexpression of TRIM16 did not reduce cell viability or proliferation in non-malignant HEK001 cells (Figures 5A and 5B). Furthermore, we examined the effect of knockdown of TRIM16 expression on cell growth. The efficiency of TRIM16 siRNA was evaluated after 72 h of transfection by western blot and demonstrated a more than 70% knockdown of TRIM16 (Figure 5C). The proliferative capacity of MET-1 and HSC-1 cells was significantly increased after transfection of TRIM16 siRNA compared with control siRNA (*p* < 0.0001) at 72 h (Figure 5C). Immunofluorescence and immunoblotting with TRIM16-specific antibodies showed significantly increased nuclear levels of TRIM16 after 24 and 48 h following transfection in MET-1 cells (Figures 5D and 5E).

We hypothesized that the effects of TRIM16 on cell growth may be mediated by effects on these cell cycle regulatory proteins in retinoid-resistant skin cancer cells. Indeed, our data here show that overexpression of TRIM16 decreased nuclear E2F1 protein expression and also markedly reduced phospho-pRb (ser807/811), compared with empty vector controls (Figures 5E and 5F). We also hypothesized that TRIM16 might directly interact with E2F1. To test this hypothesis, MET-1 cells were transiently transfected with TRIM16-GFP and E2F1-Flag plasmid DNA for 24 h. The whole cell lysates were subjected to co-immunoprecipitation (co-IP) with a GFP-tagged antibody which recognized the transfected TRIM16, and probed with an anti-Flag antibody for E2F1. Co-IP confirmed that transfected TRIM16 indeed formed a complex with E2F1 in SCC cells (Figure 5G). Significantly, ectopic overexpression of both TRIM16 and E2F1 in MET-1 cells blocked the inhibitory effect of TRIM16 on cell proliferation, compared with TRIM16-transfected cells alone (*p* < 0.0001) (Figure 5H). These results suggest that TRIM16 effects on SCC cell growth may require nuclear translocation of TRIM16 protein, where TRIM16 directly interacts with E2F1, resulting in a reduction in nuclear E2F1 and phospho-Rb levels.

### TRIM16 binds to and reduces vimentin protein expression in SCC cells

**Figure 6:**
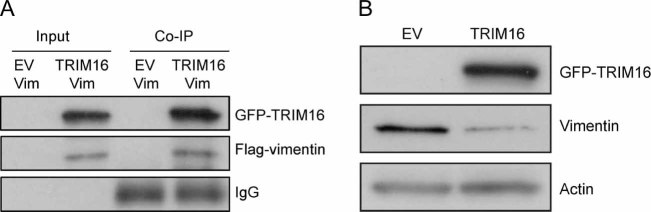
TRIM16 binds to and modulates vimentin protein expression in SCC cells. (A) Interaction of TRIM16 with vimentin. Lysates of MET-1 cells transfected with either TRIM16-GFP or vimentin-Flag plasmid DNA and immunoprecipitated with GFP antibody were then analysed by immunoblotting using anti-GFP and anti-Flag tag antibodies. (B) TRIM16 overexpression down-regulates exogenous vimentin. Lysates of TRIM16 or empty vector transfected MET-1 cells were analysed by anti-GFP and anti-vimentin antibodies. Anti-actin antibody served as a loading control

Vimentin is the predominant intermediate filament protein in mesenchymal cells and is implicated in metastasis and cancer cell migration in colon and breast cancer cell lines 21. Thus, we examined whether TRIM16 interacts with vimentin in SCC cells. Co-IP of vimentin with GFP-TRIM16 was performed on whole cell lysates from transfected MET-1 cells and confirmed that TRIM16 and vimentin formed a complex (Figure 6A). TRIM16 markedly reduced vimentin protein expression, as shown by the TRIM16 overexpression experiment in MET-1 SCC cells (Figure 6B). This finding suggests that TRIM16 overexpression may reduce cell motility through its interaction with vimentin in SCC cells.

### Overexpression of TRIM16 reduces cell motility and migration through its RFP-like domain

**Figure 7:**
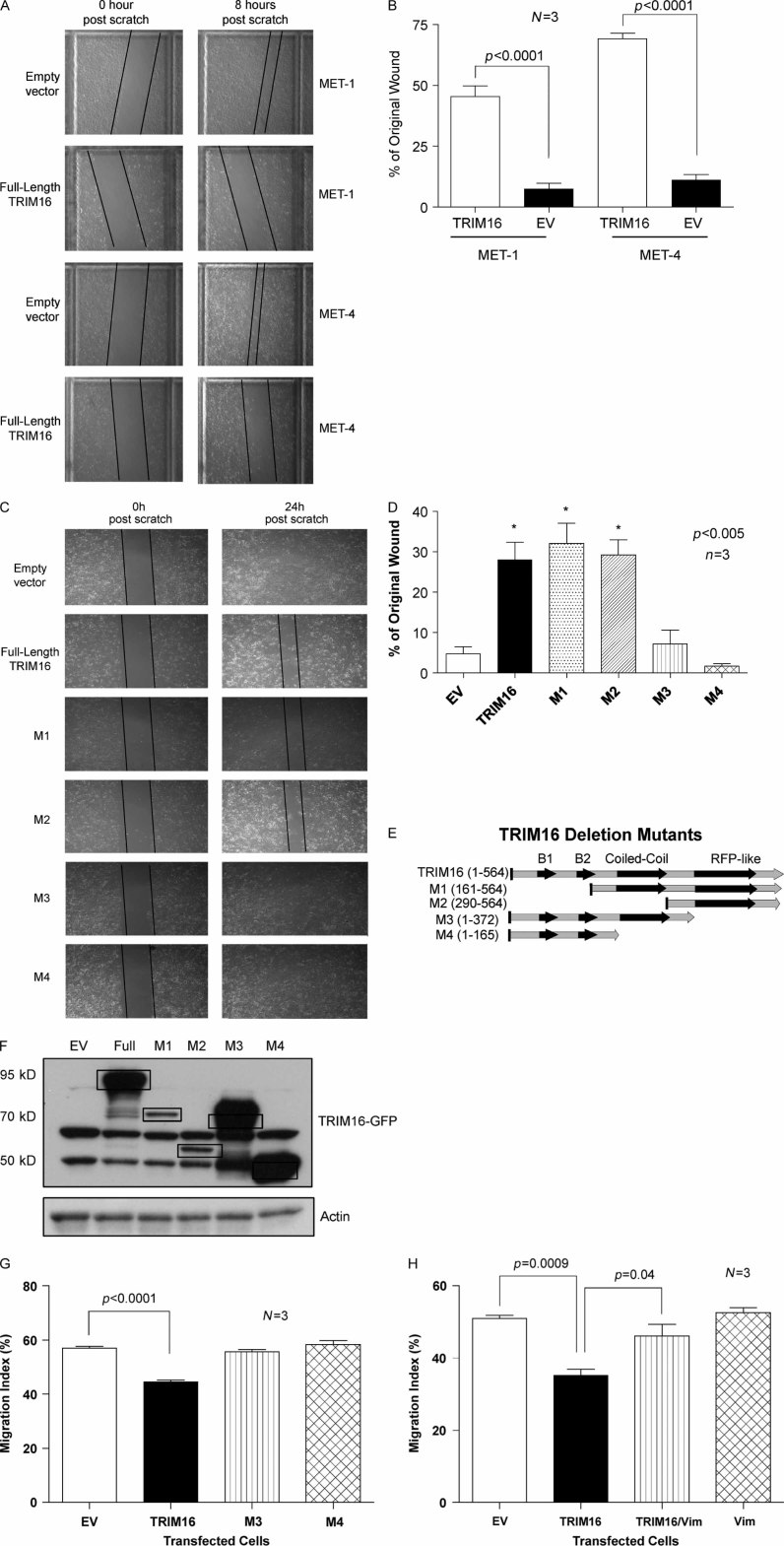
Overexpression of TRIM16 reduces cell motility and migration through its RFP-like domain. (A) Representative phase contrast micrographs of closure of scratch-wounded confluent cultures of empty vector or TRIM16 plasmid DNA transfected MET-1 and MET-4 cells after wounding and 8 h post-wounding. (B) Relative closure of MET-1 and MET-4 wounds: the average distance moved by the empty vector control and the average (and standard error) movement of TRIM16 plasmid DNA transfected cells in three independent wounds are shown relative to percentage of original wound. (C) Representative phase contrast micrographs of scratch-wounded confluent cultures of empty vector or TRIM16 full-length, M1, M2, M3, and M4 plasmid DNA transfected MET-1 cells after wounding and 24 h post-wounding. (D) Relative closure of MET-1 wounds: the average distance moved by the empty vector control and the average (and standard error) movement of TRIM16 and M1, M2, M3 and M4 transfected cells in three independent wounds are shown relative to percentage of original wound. (E) Schematic representations of TRIM16 full-length, mutant 1 (M1), mutant 2 (M2), mutant 3 (M3), and mutant 4 (M4) constructs used in this study. (F) Expression of TRIM16 full-length and deletion mutants in MET-1 cells: the lysates of MET-1 cells transiently transfected with empty vector (EV, lane 1), TRIM16 full-length (lane 2, 95 kD), mutant 1 (lane 3, 70 kD), mutant 2 (lane 4, 55 kD), mutant 3 (lane 5, 65 kD), and mutant 4 (lane 6, 45 kD) were probed with the anti-GFP tag antibody in an immunoblot analysis. (G) Invasion assay of MET-1 cells through collagen-coated cell culture inserts. The cells were transiently transfected with empty vector, TRIM16 full-length, mutant 3, and mutant 4 for 24 h. The percentage of the migrated cells divided by the total number of cells in the wells is shown. (H) Invasion assay of MET-1 cells through collagen-coated cell culture inserts. The cells were transiently transfected with empty vector, TRIM16 full-length, TRIM16 and vimentin, or vimentin alone for 24 h. The percentage of migrated cells divided by the total number of cells in the wells from three independent experiments is shown

We examined whether TRIM16 could affect cell motility in SCC cells. First, we performed a wound healing assay using transiently transfected MET-1 and MET-4 cell lines. As shown in Figures 7A and 7B, TRIM16 slowed repair of cell injury in MET-1 and MET-4 cells 8 h after wound scratch. To determine which domain of TRIM16 was required for reduced cell motility, we transiently transfected full-length TRIM16 and four different deletion mutants into MET-1 cells for 24 h. TRIM16 full-length, mutant 1, and mutant 2 slowed repair of the scratch wound. However, mutants 3 and 4 had no effect, which suggested that the effect of TRIM16 on cell motility was mediated by its RFP-like domain (Figures 7C–7E). We used immunoblot with a GFP epitope antibody to confirm that the mutant proteins were expressed in MET-1 cells following transient transfection (Figure 7F). To further investigate the role of TRIM16 in SCC cell migration, we examined the invasiveness of MET-1 cells in a Transwell plate migration assay, using 10% fetal calf serum (FCS) as a chemoattractant. As shown in Figure 7G, overexpression of TRIM16 reduced cell migration at 24 h compared with the empty vector control. In contrast, overexpression of mutants 3 and 4 did not affect cell migration at 24 h compared with the empty vector control. Significantly, ectopic overexpression of both TRIM16 and vimentin in MET-1 cells blocked the inhibitory effect of TRIM16 on wound closure, suggesting that vimentin is required for TRIM16 effects on the cell migration (*p* = 0.04) (Figure 7H). These data indicate that the overexpressed RFP-like domain of TRIM16 slows cell migration in SCC cells and suggest a specific role for TRIM16 in normal keratinocyte migration which is lost upon malignant transformation.

## Discussion

The role of TRIM16 in skin carcinogenesis has not been previously reported. For the first time, we have demonstrated that TRIM16 acts as a negative regulator in SCC growth and migration. We also showed that nuclear TRIM16 facilitates cellular retinoid sensitivity and has a role in normal keratinocyte differentiation. Thus, loss of TRIM16 function may play a role during the progression from normal skin to SCC by promoting cell division and inhibiting differentiation. Our current study reveals that TRIM16 was most strongly expressed in the more differentiated cell layers (the stratum granulosum and spinosum), compared with the less differentiated but more rapidly growing cell layer (stratum basale) in normal human skin. Overexpression of TRIM16 in SCC cell lines increased the expression level of cytokeratin 1/10 and involucrin, both of which are differentiation markers in the stratum granulosum and spinosum. These results strongly suggest that TRIM16 may play a role in terminal differentiation of keratinocytes.

In this study, we showed that TRIM16 expression was reduced in excised human actinic keratoses and further diminished in excised human SCC tumour tissue compared with normal skin. Since TRIM16 acts as a tumour suppressor, affecting cell proliferation and cell migration in different tissue types 16, 17, repressed expression of TRIM16 in SCC may contribute to the unrestrained proliferation of keratinocytes. During the early (premalignant) stages of human SCC pathogenesis, this may manifest as actinic keratoses, which can then progress to cutaneous SCC. Indeed, some authors suggest that actinic keratoses represent *in situ* cutaneous SCC 22, 23. However, the mechanism for reduced TRIM16 in actinic keratoses, and also in SCC, requires further elucidation. Further study is also needed to determine the relationship between expression of TRIM16 and clinical prognosis in skin cancer.

We have previously identified TRIM16 as a novel RARβ transcriptional regulator in the retinoid signal 15 and have also demonstrated that TRIM16 expression conferred retinoid sensitivity on many different retinoid-resistant cancer cell types 16. Our current studies of TRIM16 sub-cellular localization indicate that the protein is predominantly cytoplasmic, but retinoid treatment of sensitive normal cells (PHKs) results in nuclear translocation of TRIM16 and growth inhibition. In contrast, TRIM16 nuclear translocation was markedly reduced in retinoid-resistant SCC cells, which further suggests that loss of TRIM16 nuclear expression may contribute to retinoid resistance in skin cancer cells. However, the mechanism of TRIM16 nuclear transportation in human keratinocytes still remains to be determined. As the location of TRIM16 is associated with its role in retinoid sensitivity, growth inhibition, and cell cycle regulation, deletion mutant studies will need to be undertaken to determine which region of TRIM16 has nuclear localization potential.

Overexpression of E2F-1 in head and neck SCC cell lines has the ability to stimulate cell cycle re-entry but is also associated with increased invasiveness 24, suggesting a role in metastasis. E2F-1 is linked to the cyclin D1/p16INK4A/pRb and p14ARF/p53/mdm2 pathways, which determine whether a cell will proliferate or undergo apoptosis, respectively 25. We have recently found that TRIM16 overexpression correlated with cell growth inhibition through effects on cyclin D1 and phospho-Rb in lung cancer cells 16. Our current study reveals that TRIM16 binds directly to E2F1 and decreases nuclear E2F1 levels and cell proliferation in SCC cells. This suggests that TRIM16 may play a critical role in cell cycle progression and control cell proliferation by down-regulation of nuclear E2F1 in SCC cells.

The expression of vimentin is highly up-regulated in endothelial cells with strong migration capacity compared with endothelial cells with less migration activity 26. While the exact mechanisms of vimentin function are not yet fully elucidated, post-translational modifications of vimentin are thought to regulate the dynamic states of this protein 27. We have shown that TRIM16 bound to and modulated vimentin expression in SCC cells, reduced cell motility and migration, and that the RFP-like domain was required for TRIM16's effects on SCC cell migration, suggesting that vimentin could be used as an anti-cancer therapeutic target in SCC patients with metastatic tumours. Further studies are needed to clarify the functional interaction between TRIM16 and vimentin protein in SCC cells.

TRIM16 is a novel mediator affecting SCC growth, motility, migration, and resistance to retinoid treatment. Further studies are required to determine the mechanisms leading to low-level TRIM16 expression in SCC cells, which may ultimately lead to the development of novel compounds that can restore TRIM16 expression in human skin SCC. Our findings also highlight activation of TRIM16 nuclear translocation or degradation of vimentin and E2F1 as potential novel therapeutic strategies for the treatment of skin cancers.

## SUPPORTING INFORMATION ON THE INTERNET

The following supporting information may be found in the online version of this article.

**Supplementary materials and methods**

Transient transfection with plasmid DNA and small interfering RNA (siRNA); scratch wound assays and migration assay; viability and proliferation assays; protein half-life assay and cycloheximide treatment.
