# Supplementary material for: The retinoid signalling molecule, TRIM16, is repressed during squamous cell carcinoma skin carcinogenesis in vivo and reduces skin cancer cell migration in vitro
Source: J Pathol. 2011 Oct 18;226(3):451–62. doi: 10.1002/path.2986 (PMC3504077; doi:10.1002/path.2986)
Supplement: Supplementary file 1 [file path0226-0451-SD1.doc]

**Supporting information**

**Supplementary materials and methods**

1. *Transient Transfection with Plasmid DNA and small interfering RNA (siRNA)*

Cells (5 × 10^5^) were transfected with 3 μg pcDNA3.1(-)/TRIM16/myc-His or TRIM16-GFP plasmid DNA per 6-well plate for 24 hours before harvesting for transient transfection experiments as described [20]. SCC-15 cells were transfected using Lipofectamine 2000 (Invitrogen, Victoria, Australia) according to the manufacturer’s protocol. MET-1 and MET-4 cells were transfected with Lipofectamine LTX (Invitrogen) according to the manufacturer’s protocol.

HSC-1 and MET-1cells were seeded in 96-well plates (3 x 10^4^) for cell growth assay and in T25 flasks (1 x 10^6^) for protein analysis in TRIM16 knockdown experiments using siRNA. Cells were transfected with either 20 nM control siRNA or siRNA specific to TRIM16 (Ambion, Foster City, CA) using Lipofectamine 2000 (Invitrogen) for 24, 48 and 72 hours.

1. *Scratch wound assays and migration assay*

For scratch wound assays, MET-1 cells were transfected with either 6 μg of TRIM16-GFP full-length or TRIM16-GFP deletion mutant expression vectors or empty vector with Lipofectamine LTX, as described in our previous study [20]. For migration assays, MET-1 cells were serum starved for 2 hours and then 3 x 10^4^ cells were seeded on 0.8 µM Transwell inserts pre-coated with Collagen IV on the underside of the insert (BD Australia, Sydney). Transwell inserts were placed in companion plates containing media with 10% FCS and the cells were allowed to migrate at 37^o^C. The migration index was calculated as the percentage of the migrated cells divided by the total number of cells in the wells.

1. *Viability and proliferation assays*

Cells (1x10^4^ cells) were seeded in 96 well plates and transient transfected with either TRIM16 plasmid DNA or TRIM16 siRNA for 24 to 72 hours. Cell viability was measured using Alamar Blue cell base read out (Molecular Diagnostics, USA). Alamar Blue was added directly to the cell culture media of 96 well plates. The change of medium colour from blue to pink indicates the utilization of Alamar Blue by actively metabolic cells. This change was quantified calorimetrically on a microplate reader at 570 nm absorbance and 595 nm reference wavelengths. Absorbances of between 0.1 and 0.5 were used for accurate readings. Readings were performed 5 hours after the addition of Alamar Blue.

Incorporation of 5-bromo-2′-deoxyuridine (BrdU) was used to evaluate DNA synthesis by a Proliferation kit (Roche, Australia) according to the instructions provided by the manufacturer. Briefly, the 10,000 cells were seeded, transiently transfected at the following day. The cells were incubated with BrdU (10 μM) solution for 2 hours, fixed, then there was a 15 minute incubation with 5% FCS for blocking non-specific antibody binding before the addition of the primary antibody (Peroxidase-conjugated anti-BrdU). After washing in PBS, the peroxidase substrate was added and the absorbance of the well contents was measured on a microplate reader at 370 nm test wavelength and 490 nm reference wavelengths (Bio-Rad, Australia). Wells with only media were used as blank controls.

1. *Protein half-life assay and cycloheximide treatment*

MG132 (Biomol, USA) was used to inhibit protein degradation via the proteasomal degradation pathway. 80% confluent cells were treated with MG132 at 30 μM for 5 hours, directly before harvest. Cells (3x10^5^ cells) were seeded in 6 well plates and treated with 100mg/ml cycloheximide (Biomol) over a period of 2 to 8 hours.

1. *Nuclear and cytoplasmic protein extractions*

Cells (1x10^6^ cells) were seeded in T75 tissue culture flasks and transient transfected with either TRIM16-GFP plasmid DNA or GFP empty vector plasmid for 24 to 48 hours. Cells were harvested at each time point and the nuclear and cytoplasmic proteins were isolated using NE-PER Nuclear and Cytoplasmic Extraction Kit (Thermo Scientific, Australia) according to manufacture protocol. Extracted proteins were quantified with a BCA protein quantitation kit (Thermo Scientific).

**Reference**

**(Note: reference number corresponds to reference list in main article)**

20. Breitkreutz D, Stark HJ, Plein P, *et al.* Differential modulation of epidermal keratinization in immortalized (HaCaT) and tumorigenic human skin keratinocytes (HaCaT-ras) by retinoic acid and extracellular Ca2+. *Differentiation* 1993; **54:** 201–217.
